# Molecular Insight into Prostate Cancer: Preventive Role of Selective Bioactive Molecules

**DOI:** 10.3390/life13101976

**Published:** 2023-09-27

**Authors:** Mohd Jameel, Homa Fatma, Liudmila A. Nadtochii, Hifzur R. Siddique

**Affiliations:** 1 Molecular Cancer Genetics & Translational Research Lab, Section of Genetics, Department of Zoology, Aligarh Muslim University, Aligarh 202002, Indiahfatma@myamu.ac.in (H.F.); 2 Department of Microbiology, Saint Petersburg State Chemical & Pharmaceutical University, 197022 Saint Petersburg, Russia

**Keywords:** prostate cancer, chemoresistance, metastasis, chemopreventive phytochemicals, signaling pathways

## Abstract

Prostate cancer (CaP) is one of the most prevalent male malignancies, accounting for a considerable number of annual mortalities. However, the prompt identification of early-stage CaP often faces delays due to diverse factors, including socioeconomic inequalities. The androgen receptor (AR), in conjunction with various other signaling pathways, exerts a central influence on the genesis, progression, and metastasis of CaP, with androgen deprivation therapy (ADT) serving as the primary therapeutic strategy. Therapeutic modalities encompassing surgery, chemotherapy, hormonal intervention, and radiotherapy have been formulated for addressing early and metastatic CaP. Nonetheless, the heterogeneous tumor microenvironment frequently triggers the activation of signaling pathways, culminating in the emergence of chemoresistance, an aspect to which cancer stem cells (CSCs) notably contribute. Phytochemicals emerge as reservoirs of bioactive agents conferring manifold advantages against human morbidity. Several of these phytochemicals demonstrate potential chemoprotective and chemosensitizing properties against CaP, with selectivity exhibited towards malignant cells while sparing their normal counterparts. In this context, the present review aims to elucidate the intricate molecular underpinnings associated with metastatic CaP development and the acquisition of chemoresistance. Moreover, the contributions of phytochemicals to ameliorating CaP initiation, progression, and chemoresistance are also discussed.

## 1. Introduction

Prostate cancer (CaP) develops from the unregulated proliferation of prostate cells triggered by various genetic and epigenetic alterations. CaP is the predominant form of cancer in males, impacting men worldwide [1]. Based on GLOBOCAN, 2020 witnessed around 1.41 million fresh incidences of CaP diagnosed on a global scale, and CaP ranked as the second most prevalent cancer and ranked fifth in cancer-related mortalities among men [2]. Interestingly, when diagnosed at the initial stage, the 5-year survival rate of CaP patients is high; however, a significant drop in the overall 5-year survival rate has been observed in patients diagnosed with distant metastasis [3]. The majority of reported CaP cases are adenocarcinomas. However, other variants, such as sarcomas, small cell carcinomas, neuroendocrine tumors, and transitional cell carcinomas, have also been documented [4,5]. The risk factors include lifestyle shifts, dietary habits, reduced physical activity, environmental influences, smoking, socioeconomic disparities, ethnicity, vitamins, mineral supplements, alcohol, and sex hormones. Moreover, studies highlight a direct correlation between the Human Development Index (HDI) and CaP incidence and mortality [2,6].

Prostate-specific antigen (PSA) is a serine protease found in prostate epithelial tissues, one of the most widely used biomarkers for the early screening and detection of CaP. Other biomarkers based on serum, urine, and tissues are also being incorporated as diagnostic and prognostic tools [4,7,8]. The interaction of androgens with androgen receptors (ARs) is important for normal cell as well as CaP cell growth and proliferation [9]. The AR regulates the expression of PSA through androgen response elements (AREs) and the recruitment of primary and secondary coactivators [10]. Possible treatment options for CaP depend upon the risk category. Men in the low-risk CaP category choose active surveillance, and those in the intermediate-risk category opt for ablative surgery or radiation therapy. However, high-risk patients are treated with radiation and hormonal therapy. Further, to slow down tumor progression in the advanced metastatic stage, androgen deprivation or suppression therapy (AST) with or without chemotherapy is often employed [11]. Irrespective of the initial successful outcomes seen with AST, patients eventually develop resistance to chemotherapy, progressing towards castration-resistant prostate cancer (CRPC). CRPC is a disease condition where cancer progresses independently of the androgen levels in the body. The FDA has approved multiple treatment options for CRPC conditions, such as Abiraterone, Enzalutamide, Docetaxel, Cabazitaxel, and Sipuleucel-T (Sip-T). However, these treatments typically extend median survival by 18 to 24 months [12].

Chemoresistance is the capability of cancerous cells to escape or survive even in the presence of therapeutics. CaP chemoresistance is positively linked with aggressiveness and metastasis. Notably, resistance in CRPC involves cellular resistance mechanisms in CaP cell subpopulations, impaired drug delivery, and a negligible interaction between CaP cells and their microenvironment [13]. Various strategies to chemosensitize resistant cells focus on targeting mechanisms that are responsible for the therapy resistance, such as the ATP binding cassette (ABC) transporter proteins. Among these, ABCB1 (a steroid transporter) has been extensively investigated, revealing its significant implication in the progression of this disease [14]. Several investigations have indicated that the ABCB1 transporter is downregulated in CaP due to the hypermethylation of its promoter, particularly when compared to adjacent non-malignant tissues, leading to the accumulation and continuous accumulation of androgens. Moreover, it was observed that CaP cells acquire resistance by overexpressing anti-apoptotic and stress-related proteins while downregulating pro-apoptotic proteins [4,15,16]. Several research labs have investigated the use of phytochemicals as chemosensitizing agents. The multitargeted properties and negligible toxic effects make phytochemicals important agents to explore for their anticancer and chemosensitizing properties [17,18]. This review discusses the underlying molecular mechanism in CaP initiation, progression, and reprogramming. Moreover, we also discuss the role of a few phytochemicals in CaP prevention and chemosensitization.

## 2. Prostate Cancer Progression and Metastasis

**Figure 1:**
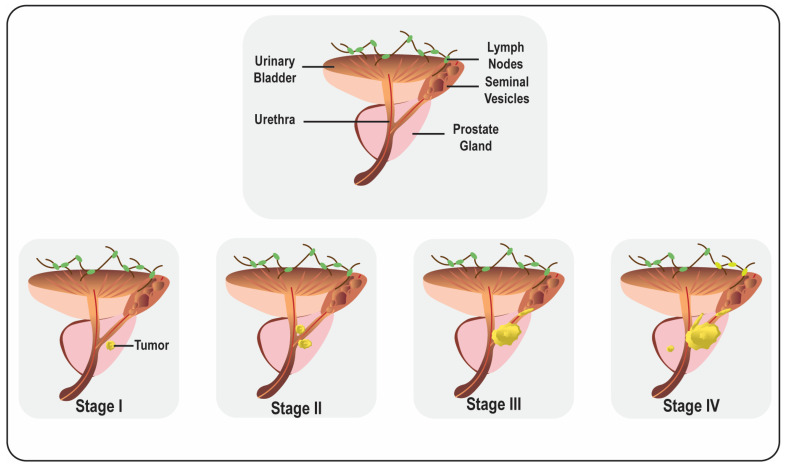
Representative diagram of male reproductive organs and prostate cancer progression.

CaP is generally divided into localized, locally advanced, and metastatic CaP [19]. At the advanced stage, CaP spreads from the prostate to different body parts (Figure 1). Usually, metastasis occurs through the lymphatic route to the pelvic and para-aortic lymph nodes and hematogenously spreads to the skeletal system, predominantly affecting the bones. It has also been noticed that visceral metastasis to the liver, lungs, and organs is comparatively rare and connected with intermittent pathology and a poor prognosis [20].

The AR signaling pathway is central to prostate carcinogenesis and advances towards androgen-independent states. Almost all primary CaP cases exhibit some level of expression of the AR. Abnormal AR signaling can be associated with a rising serum PSA level, which is treated as the prognostic marker for CaP patients [21,22]. Hormone therapy in the form of medical or surgical castration remains the mainstay of systemic treatment for CaP. Despite an initial favorable response to hormone therapy, these tumors will develop androgen independence over time, resulting in death. Although AR is central to the initiation and growth of CaP, various other pathways are also involved in CaP progression and metastasis [23,24].

### 2.1. Prostate Cancer and Androgen Receptor Signaling Pathway

The AR is an androgen-activated steroid hormone receptor belonging to the nuclear receptor superfamily. The function of the AR is dependent on its ligand, and the interaction between the AR and androgens (testosterone and its active form, dihydrotestosterone) is essential for the normal development and function of male reproductive organs. The interaction of the AR and androgens results in the separation of Hsp90 from the AR, facilitating the formation of AR nuclear translocation [9,25,26]. However, the deregulated expression of the AR or androgens results in CaP development and progression. Early-stage CaP cells are AR-positive, while late-stage CaP cells are undifferentiated and AR-negative [21]. The deregulated AR signaling axis also deregulates the expression of target genes such as PSA, fibroblast growth factor 8 (FGF8), cyclin-dependent kinase 1 (CDK1), transmembrane serine protease 2 (TMPRSS2), and prostate transmembrane protein androgen induced 1 (PMEPA1), resulting in CaP progression, development, and metastasis [27]. The number (19–25 repeats) of Glutamine (CAG) repeats in the N-terminal transactivation domain affects the transcriptional activity of the AR. It was observed that shorter Glutamine repeats result in higher AR transcriptional activity and is associated with an increased CaP risk [28]. Moreover, histone methyltransferase DOT1L was observed to be overexpressed in CaP and upregulates the AR via c-MYC by suppressing c-MYC-regulated E3 ubiquitin ligases like HECTD4 and MYCBP2 [29].

Further, the AR helps in CaP cell growth and survival by modulating autophagy-related genes. Studies suggest that autophagy has the central oncogenic role in CaP progression, where transcription factor TEFB and four autophagy genes, such as autophagy-related 4B/4D cysteine peptidase (*ATG4B* and *ATG4D)* and Unc-51-like autophagy-activating kinases 1 and 2 *(ULK1* and *ULK2),* play crucial roles in CaP cell survival, proliferation, invasion, and migration [30]. The AR is associated with the co-activator NCOA2 and facilitates CaP cell proliferation and metastasis through the modulation of the PI3K and MAPK/ERK axis. Further, the androgen-dependent activation of ERK1/2 regulates the functional activity of transcription factors independent of AR-DNA binding and increase the level of c-FOS by activating the ETS domain-containing protein Elk-1. Interestingly, the interaction of the AR with p85a induces AKT kinase activity via phosphatidylinositol-3,4,5-triphosphate (PIP3), signaling lipid generation and the activation of the p110 subunit [28,31]. Another study reported that FOXA1, GATA2, and OCT1 can increase the AR-DNA interaction and level of FOXA1, positively correlates with CaP stages, Gleason scores, and CaP-specific survival [32]. AR signaling in the stroma of the prostate is also essential for the development of carcinogenesis. During normal prostate development, androgen promotes mesenchymal paracrine secretion; hence, the increased paracrine secretion of stimulatory growth factors due to androgen action might also result in accelerated CaP progression [33]. Further, AR-V1, AR-V7, and AR-V567es are overexpressed in CRPC bone metastasis compared to hormone naïve bone metastasis and are associated with poor survival [30]. Although the depletion of androgens via androgen ablation temporarily represses tumor growth, most CaP patients eventually develop androgen-insensitive tumors, for which no effective therapy exists [21].

### 2.2. WNT/β-CATENIN Signaling Pathways

WNT/β-CATENIN signaling is essential for organogenesis during embryonic development; however, the alteration of adult cells leads to cancer formation. WNT signaling and β-CATENIN play important roles in the various oncogenic processes that give rise to different human malignancies, including CaP [34,35]. WNT/β-CATENIN signaling mediates upsurges in receptor Tyrosine kinase-like orphan receptor 1 (ROR1), increasing noncanonical responses to Wnt5a, which plays an important role in prostate stem cell generation during early development and might be reactivated in the CaP microenvironment. The upregulation of the WNT transporter Wntless (WLS) tumor cells was strongly linked with the activity of the WNT/β-CATENIN pathway in primary CaP along with CRPC [36,37]. Protein Kinase D1 (PrKD1) is known to be a unique serine/threonine kinase that causes the phosphorylation of Threonine120 (T120) of β-CATENIN, consequently elevating the nuclear β-CATENIN level in CaP. Moreover, it was observed that β-CATENIN, along with the MYC and MAX heterodimers, bind to the PrKD1 promoter region and repress its activity in CaP cells, suggesting that β-CATENIN/c-MYC/MAX downregulate PrKD1 through an autorepressive feedback loop [38].

Interestingly, WNT/β-CATENIN signaling also drives Cap-mediated bone metastasis. The tumor cell at the primary site usually develops mesenchymal properties, which were reported to be regulated by FZD4, a WNT receptor. WNT signaling, particularly Wnt5A, is very important as the cells flow in the bloodstream. The cells, after reaching the bones, retain the tumorigenic or stem-like property, which is supported by the WNT or β-CATENIN signaling and involves CHD11, CD24, and Wnt5A for CaP-mediated bone metastasis progression [39]. WNT-11 belongs to the WNT protein family and is a part of the non-canonical signaling pathway. It was theorized that WNT-11 is overexpressed in CaP cells and is involved in CaP progression and migration and the secretion of factors such as neurone-specific enolase to differentiate CaP cells in neuroendocrine-like cells [40]. The gene amplification and overexpression of transcription factor SOX-2 plays an important role in cancer progression and the metastasis of various cancers, including CaP. Interestingly, SOX-2 modulates EMT via the WNT/β-CATENIN signaling pathway [41].

### 2.3. Prostate Cancer and Phosphoinositide 3-Kinase Pathway (PI3K)/AKT

Abnormality in the PI3K pathways is recorded in the different human cancer types, especially in advanced CaP cases. The role of PI3K in CaP has been well recorded, and it was shown that PI3K-C2β, the isoform of class II PI3Ks, regulates the activation of extracellular signal-regulated kinase (ERK1/2) and mitogen-activated protein kinase (MEK1/2) and mediates the invasion and migration of CaP cells [42,43]. It was observed that the activation of the serine/threonine kinase, AKT, can lead to CaP cell proliferation through the MAPK pathway. MAPK4 activates AKT via the Caspase 4/5-dependent pathway and represses the degradation of GATA2, which concurrently activates the AR [44]. Moreover, CaP progression has been linked with PTEN loss and AKT-dependent hexokinase, as reprograming the glycolytic pathway can improve the overall outcome [45]. Further, the N-Cadherin-mediated dysregulation of monocyte chemoattractant protein-1 stimulates PI3K/AKT signaling, which targets NF-kB, leading to the stimulation of the bone morphogenetic protein-1 (BMP) signaling cascade. The PI3K/AKT/NF-kB/BMP/SMAD axis results in CaP invasion and bone metastasis [46].

It was reported by Mahmoud et al. [47] that the inhibition of AKT via the increased expression of ULK1 mRNA and the LC3II protein can significantly decelerate CaP progression, suggesting the role of AKT in CaP development. In addition, the inhibition of the key molecules of the PI3K/AKT pathways causes blockage in CaP cell proliferation and metastasis via Cyclin D1 downregulation along with the upregulation of p27. An epithelial cell adhesion molecule (EpCAM) upregulates E-Cadherin, the PI3K/AKT/mTOR signaling axis, p-4EBP1, and p-S6K1 expression to promote CaP proliferation. A decrease in tumor sphere formation and CaP progression was observed in CaP cells when EpCAM was targeted, with a significant decrease in PI3K/AKT/mTOR expression, suggesting that EpCAM can be the therapeutic target to inhibit CaP progression [48,49]. Further, hypoxic conditions promote the PI3K/AKT/mTOR pathway in CaP cells for the adaptation of cells to a low O_2_ microenvironment via hypoxia-induced factor 1 (HIF-1) expression, suggesting the role of the PI3K/AKT/mTOR axis in CaP during both normoxic and hypoxic conditions [50].

A reduction in the expression of the PLK4 and PI3K signaling molecules in CaP cells can suppress cell proliferation, migration, invasion, and metastasis [37]. c-MYC is the oncoprotein that has been implicated in various cancers, including CaP [51]. It was reported that the level of c-MYC is relatively elevated in metastatic CaP. c-MYC directly promotes CaP development through the upregulated expression of various pro-tumorigenic factors, including ribosome biogenesis, which supports tumor growth and PI3K/AKT/mTOR pathway dysregulation, consequently promoting the survival and growth of CaP cells. The c-MYC/PI3K/AKT/mTOR axis can be targeted to decrease tumor progression [52].

### 2.4. Prostate Cancer and JAK/STAT Pathway

The JAK/STAT signaling pathways are found to be continuously activated in different types of malignancies, including CaP. This signaling pathway in CaP is associated with tumor growth, upregulated angiogenesis, and metastasis [42,53]. It was reported that JAK/STAT pathway activation drives prostate tumor plasticity. Interestingly, the role of cytokines in cell proliferation and migration is well known, especially via cytokines. Within the different cytokines, interleukin-6 (IL-6) greatly impacts CaP development and regulates cell growth and immune response through the activation of the JAK/STAT signaling pathways along with cross-talk with PI3K, MAP kinase, and different cellular pathways [54]. In CaP cells, BRCA1 and the AR can bind to STAT3, leading to the activation of the downstream JAK/STAT signaling pathway and upregulating anti-apoptotic and angiogenic proteins such as Bcl-xl, VEGF, Survivin, BCL-2, and MCL-1 [55]. Moreover, it is also well observed that the frequent over-activation of the JAK/STAT pathway in CaP cells can initiate the upregulation of PD-L1. Interestingly, STAT3 plays an important role as a mediator of tumor immune evasion. It was shown that the SOCS3 gene of the adenoviral vector increases the sensitivity of CaP via the over-activation of JAK/STAT3 to NK cells via the decline in PD-L1 expression and the generation of IL-6 [56].

### 2.5. Other Factors Involved in Prostate Cancer

MicroRNAs (miRNAs), small noncoding RNAs, play important roles in CaP progression and metastasis. Numerous studies showed that miRNA acts as either a promoter or inhibitor of metastasis in CaP. The dysregulation of the miRNA-34 family was reported in different cancer types, including CaP. Due to their interaction with the tumor suppressor p53 and their participation in EMT via EMT-TFs, miR-34 family members are known as tumor-suppressive miRNAs. By directly suppressing CD44, miR-34a prevents metastasis [57]. Moreover, the failure of miR-34a expression stimulates the PI3K/AKT, JAK/STAT3, and WNT/β-CATENIN signaling pathways, leading to the progression of CaP [58]. Deregulated miRNA can be used as the novel diagnostic and prognostic tool in CaP as there may be a relationship between the patterns of miRNA expression and the androgen dependence of these tumors, indicating a potential link between miRNA expression and the tumor’s reliance on androgens for growth. Further, the downregulation of miR-34a promotes CaP progression by downregulating the oncoprotein STMN1 via the CtBP1\miR-34a\STMN1\GDF15 axis. Moreover, the MiR-34b plays a critical role in migration and invasion inhibition by regulating the TGF-β pathway [59]. Three urine microRNAs (miR-21-5p, miR-141-3p, and miR-205-5p) were observed to be promising non-invasive diagnostic markers for CaP. Additionally, it was found that miR-21-5p, miR-574-3p, and miR6880-5p were significantly elevated in patients with CRPC, suggesting their potential as prognostic biomarkers for CRPC. Specifically, the increased levels of miR-21-5p were associated with the downregulation of programmed cell death protein 4, a regulator of prostate cancer cell growth and resistance to castration. On the other hand, elevated miR-574-3p levels were linked to the suppression of the Notch signaling pathway, as well as changes in DNA damage and apoptosis [60]. By measuring substances called 8-OHdG and 8-Iso-PGF2α in the urine of prostate cancer patients, researchers have discovered consistent and significant associations between increased levels of oxidative stress indicators and CaP. Additionally, they found that when radical prostate cancer removal surgery is performed, these indicators tend to return to normal levels. Even though these biomarkers are not highly specific for prostate tissue and diagnosis, the findings suggest that assessing 8-OHdG and 8-Iso-PGF2α in urine, both before and after surgery, could serve as a valuable technique for predicting the success of radical prostate cancer surgery and possibly for identifying the risk of local recurrence following the procedure [61].

CaP metastasis involves complex interplay and cross-talk between different molecules. It is noted that a high level of Plectin plays an important role in the development of localized and metastatic human CaP, and its knockdown slows the growth of CaP and impairs aggressive cellular behaviors [62]. Various LNCaP cell subpopulations showed a non-integral copy number and a high mutational load. The DNA mismatch repair genes in all three cell lines had pathogenic mutations, homozygous deletions, harmful alterations affecting the cell cycle, and other vital cellular functions [63]. Moreover, calcium is also important in controlling the molecular factors and signaling pathways involved in CaP metastasis. Calcium channels are generally present at the membrane region of the endoplasmic reticulum (ER), like IP3 receptors, and are reportedly involved in CaP cells’ survival. The dysregulation of the calcium channel and its receptor may also lead to CaP progression and metastasis [64]. Moreover, NF-κB transcription factor members are crucial mediators of CaP progression, migration, and invasion. It was also reported that NF-κB activity is higher in metastatic CaP compared to localized disease [8,22]. Studies have shown that the inhibition of IRE1α by MKC8866, an IRE1α RNase-specific inhibitor, strongly inhibits CaP tumor growth. Interestingly, a global transcriptomic analysis has revealed that IRE1α-XBP1s pathway activity is mandatory for c-MYC signaling, which is upregulated in CaP development [65].

Similarly, it was also observed that immunoglobulins are very crucial for the regulation of different cancers, including CaP. The level of IgG1 heavy chain was found to be elevated in CaP patients. The activation of IGHG1 via antibody blocking or genetic knockdown resulted in the suppression of cell growth, the initiation of cell cycle arrest, and, finally, apoptosis. There was a positive correlation between the level of c-MYC and IGHG1. Moreover, MEK/ERK/c-MYC pathways are observed to be downstream of IGHG1 in CaP cells. Further, it was observed that IGHG1 inhibition controlled the tumor growth and inactivation of the MEK/ERK/c-MYC pathway [66]. Gaining a deeper understanding of how different signaling pathways interact and influence the progression of CaP is critically important. This knowledge is pivotal for the development of targeted medications that can inhibit or activate specific molecules, ultimately offering the potential to regulate tumor advancement. Further, many deregulated pathways can serve as diagnostic or prognostic markers for CaP. Therefore, it is essential to have a comprehensive understanding of the signal cascades that underlie the initiation and development of CaP. This knowledge is crucial for the eventual development of effective strategies to combat CaP.

## 3. Current Prostate Cancer Therapy

CaP onset and progression are closely related to the activation of androgen-dependent signaling pathways. Targeting these pathways has been the main approach for treating metastatic CaP using AR pathway inhibitors [67]. Over the past 30 years, several anti-AR drugs have been developed and approved for various stages of CaP. The first-generation AR antagonists approved by the FDA include Nilutamide, Bicalutamide, and Flutamide. The availability of second-generation AR antagonists, starting with the approval of Enzalutamide by the FDA in 2012, has expanded the options for treatment. Subsequently, Apalutamide and Darolutamide were approved in 2018 and 2019, improving the survival rates in CaP patients. However, while these drugs provide prolonged survival benefits, the eventual development of resistance has been reported [68,69]. Disease progression and the restoration of AR signaling play critical roles in the progression of CRPC, and the amplification of the AR gene is a common molecular change associated with the development of CRPC. Researchers and clinicians are actively developing second-generation AR antagonists with a higher binding affinity and specificity to target AR signaling in CRPC patients. Understanding the molecular mechanisms of AR overexpression and amplification is crucial for developing novel therapeutic approaches to improve outcomes in CaP patients [70,71]. Among the different FDA-approved second-generation AR antagonists is Enzalutamide (MDV3100), which is the first second-generation drug that was developed for CRPC treatment and shows too much binding affinity towards AR compared to first-generation AR antagonists. It was observed that Enzalutamide significantly delays the overall survival and metastatic-free survival of CRPC patients and was approved by the FDA for use in patients in the year 2019. Even though Enzalutamide is extensively used in the clinical treatment program for CRPC and castration-sensitive CaP (CSPC), the increased level of Enzalutamide in the brain is associated with CNS-related events like seizures, as it can antagonistically bind to the GABAα receptor and cause multiple toxic effects in other organs [69,72]. Other AR antagonists, such as Apalutamide, which has a lower capacity to cross the blood–brain barrier, a common core structure like Enzalutamide, and fewer brain seizure side effects, were developed as an alternative [73]. It was observed that Apalutamide is known to have a significant increase in the metastasis-free survival rate of nonmetastatic CRPC in addition to the overall survivorship of metastatic CSPC [74,75]. Moreover, the recently approved second-generation AR antagonist, Darolutamide, shows less potential for CNS side effects as it cannot cross the blood–brain barrier. A clinical trial has shown that Darolutamide has good antitumor properties with fewer side effects than Enzalutamide and Apalutamide [76]. However, the long-term use of AR antagonists causes drug resistance, and the clinical benefits have been attenuated very rapidly, which generated motivation in the scientific community to develop new AR antagonists or any other alternative to the therapeutic approach. Potential new treatment options for CRPC include prostate-specific membrane antigen (PSMA) ligand therapy and newer targeted drugs such as PARP inhibitors. Moreover, different clinical trials have used a combination treatment approach with immunotherapy. Future CaP treatments will be more specifically personalized based on the findings of molecular investigations of tumor tissue, circulating tumor cells, and DNA [77].

Generally, for so many decades, it was thought that CRPC was non-treatable. However, significant life-prolonging effects were noticed in five available drugs: Cabazitaxel Abiraterone, Docetaxel, Enzalutamide, and Radium-223. The AR targeting agents like Enzalutamide and Arbiraterone considerably prolong the overall survival rate before and after therapy with Docetaxel. Moreover, Cabazitaxel could be applied as a secondary option to Docetaxel. Furthermore, Radium-223, an α emitter, could be included in the third line of therapy for symptomatic patients with bone-limited disease only. Apalutamide, Darolutamide, and Enzalutamide significantly lengthen the metastasis-free life in individuals with castration resistance and result in a quick PSA doubling time but without metastases in conventional imaging. There are promising new treatment options for CRPC, including PSMA ligand therapy and newer targeted drugs such as PARP inhibitors [78]. Clinical trials are presently evaluating treatment plans that combine immunotherapies. In the future, CaP treatment will become more and more individualized to the findings of the molecular investigations of tumor tissues, circulating tumor cells, and DNA.

## 4. Chemoresistance: A Hurdle of Current Therapy

**Figure 2:**
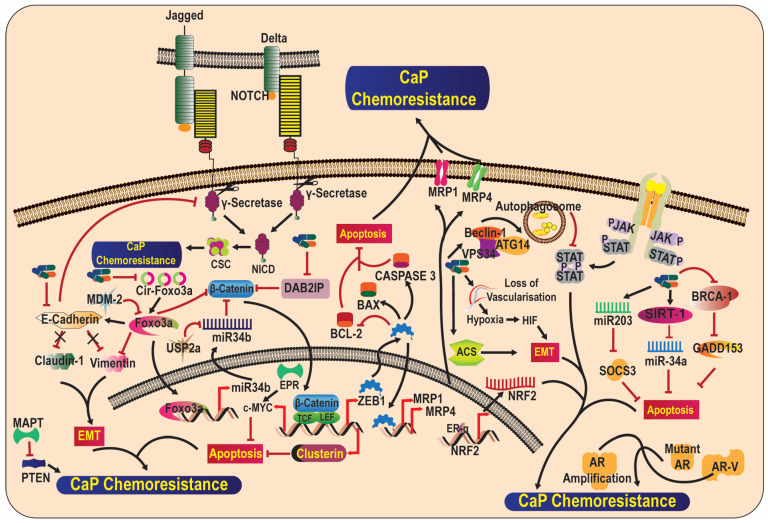
Signaling pathways involved in therapy resistance of prostate cancer. Various pathways are implicated in the development of therapy resistance in prostate cancer, including androgen-independent activation of androgen receptors, facilitation of epithelial–mesenchymal transition, and anti-apoptotic pathways. ACS: Acetyl-CoA Synthetase; AR: Androgen Receptor; AR-V: Androgen Receptor Variant; ATG14: Autophagy-Related 14; DAB2IP: Disabled Homolog 2-InteractingPprotein; EMT: Epithelial–Mesenchymal Transition; FOXO: Forkhead Box; GADD153: Growth Arrest and DNA Damage-Inducible Gene 153; JAK: Janus Kinase; LEF: Lymphoid Enhancer Factor; MDM: Murine Double Minute 2; miR: MicroRNA; MRP: Multidrug Resistance Protein; NICD: Notch Intracellular Domain; SIRT1: Sirutin; STAT: Signal Transducer and Activator of Transcription; USP2A: Ubiquitin-Specific Peptidase 2a; TCF: T-Cell Factor; VPS34: Vacuolar Protein Sorting 34; ZEB: Zinc Finger E-Box-Binding Homeobox.

The early detection and diagnosis of CaP leads to early surgical resection and the introduction of anticancer agents. The early intervention of the therapeutic regimen leads to an overall increase in the survival rate and a decrease in CaP-related mortality. However, most patients develop castration-resistant CaP after 2–3 years of routine treatment. One of the reasons for CaP chemoresistance is tumor cell heterogeneity, indicating that human cancers have a hierarchal organization and very few cells are composed of tumor-initiating as well as tumor-propagating cells [79]. Many factors, such as transcription factors, miRNA, and signaling pathways, contribute to CaP chemoresistance (Figure 2). Usually, CaP malignancy depends on the androgen signaling pathway, and androgen suppression therapy can help to prevent tumor growth. However, the majority of CaP patients develop androgen independence via abnormal androgen receptor signaling, which promotes tumor growth [40].

A resistance to ADT can lead to the development of terminal CRPC, where cancer cells become independent of androgen and are associated with an increased mortality. Various hypotheses are put forward to explain ADT, including the emergence of CSC, which further results in a heterogenous tumor microenvironment, resistance, and recurrence. Interestingly, ADT can result in a loss of vascularization, leading to hypoxia. A positive correlation was observed between the failure of ADT and the loss of vascularization over time, which subsequently resulted in decreased oxygen levels inside the tumor microenvironment, the activation of HIF, and ultimately, EMT-related genes, tumor progression, and therapy resistance [80]. Konoshenko et al. [81] performed a meta-analysis on the involvement of miRNA in CRPC development and ADT sensitivity. The authors observed that miR-23b, miR-21, miR-27b, miR-125b, and miR-221 regulate ADT resistance and have the potential to predict the response of ADT. In another study, it was observed that prostate-tumor-associated macrophages can mediate ADT response. The recruitment of macrophages enhanced Gas6 secretion, resulting in the subsequent overexpression of RON and Axl receptors, ultimately facilitating CRPC [82]. The estrogen receptor-α (ER-α) is also implicated in the precancerous stage, advanced CaP, and CRPC. It was reported that following ADT, CaP cells can shift to ER-α signaling in order to maintain CaP cell growth and proliferation even sans androgen signaling. In Bicalutamide resistance, advanced CaP cells were observed to express the ER-α–nuclear factor E2-related factor 2 (NRF2) signaling axis, where ER-α binds to the NRF2 promoter through the estrogen response element, consequently increasing NRF2 mRNA expression. The ER-α-NRF2 signaling pathways activate the Bicalutamide resistance gene [83]. Further, the microtubule-associated protein tau (MAPT) is linked with a poor overall outcome and showed an inverse correlation with PTEN. Interestingly, patients with overexpressed MAPT showed an increased resistance to Bicalutamide [84]. In another study, Sekino et al. [85] observed that Bicalutamide resistance is also associated with Protocadherin B9.

cAMP response element binding protein 5 (CREB5) mediates the activity of AR by regulating various promoters and enhancers involved in the cell cycle and proliferation, including c-MYC. The interaction of FOXA1 with CREB5 facilitates Enzalutamide resistance. Further, the increased expression of c-MYC via erythropoietin-producing human hepatocellular receptors is critical for Enzalutamide resistance. Thus, suppressing FOXA1/CREB5 interaction or c-MYC can be the possible therapeutic strategy to inhibit CRPC [86]. A marked increase in the expression of ARV7 was observed following Enzalutamide treatment. Interestingly, the knockdown of ARV7 can resensitize resistant CaP cells for Enzalutamide, suggesting the crucial role played by the ARV7 in promoting resistance [87]. He et al. [88] proposed that non-canonical AR binding sites that lack normal response elements and FOXA1 binding sites drives CaP resistance to Enzalutamide. The non-canonical AR binding site has enhanced the AR binding intensity with the CpG island, the binding sites for CXXC5 (unmethylated CpG dinucleotide-binding protein), and TET2. Enzalutamide-resistant CaP has overexpressed CXXC5 and the downstream gene *ID1* and has enhanced H3K27ac modification.

AR activation through various AR alterations is commonly observed during CaP therapy resistance. AR gene mutations, amplifications, and overexpression; extragonadal androgen synthesis; and the continuous activity of AR variants (AR-Vs) are implicated in CaP resistance [71]. Almost 20 ARVs have been identified and implicated in CaP-resistant cells. ARv7 drives progressive phenotypes in the absence of canonical AR signaling. Coregulators such as HOXB13 and ZFX can regulate the function of ARv7 during chemoresistance [89]. The presence of ARv7 in the initial stages of CaP has been associated with an unfavorable prognosis after radical prostatectomy, and its expression is frequently elevated in the tumors of castration-resistant prostate cancer (CRPC). Research indicates that these AR splice variants enhance the functioning of genes related to androgen metabolism, providing cells with a survival edge in conditions of limited androgen availability [21]. In contrast to high stromal AR expression, which facilitates CaP cell progression, low stromal AR expression is associated with biochemical relapse and disease progression [90].

BRCA1 mutation is associated with aggressive CaP progression and Doxorubicin chemoresistance. BRCA1 modulates GADD153 in p53-mutated CaP cells, targeting DNA damage response and cell cycle regulation [91]. In p53-mutated Du-145 and p53-null cells, the expression of miR-34a is greatly reduced with the increased transcription of SIRT-1, causing Camptothecin chemoresistance and decreased CaP death. It was observed that the ectopic expression of miR-34a in p53-attenuated cells inhibits cancer growth, cell cycle arrest, and decreased Camptothecin chemoresistance [92]. Li et al. [93] observed that exosome-derived miRNAs have a pleiotropic function in the development of chemoresistance. A study on the exosome regulatory network and CaP chemoresistance linkage revealed that exosome miRNAs regulate AR, PTEN, and TCF4 genes in chemoresistant cancer cells. miR-203 is highly upregulated, and miR-34b is downregulated in the exosomes derived from CaP cells. The ubiquitin-specific protease 2a-mediated downregulation of miR-34b exerts chemoresistance via c-MYC. In another study, it was found that the coordinated activation of AKT and GATA2 by MAPK4 leads to the proliferation, progression, and development of CRPC [44]. In another study, the oncogenic activation of the PI3K/AKT signaling pathway, along with epigenetic anomalies, are the properties of CRPC. It is well known that Ubiquitin-like containing PHD Ring Finger 1 (UHRF1) acts as a key epigenetic regulator and plays a critical role in the development of CaP. The overexpression of UHRF1 and p-AKT in Abiraterone-resistant CaP cells was recorded [94].

DOC-2/DAB2 interactive protein (DAB2IP) modulates various signaling pathways and suppresses metastasis by suppressing cell growth and survival and inducing apoptosis. The downregulation of DAB2IP in CaP cells leads to radio- and chemoresistance. DAB2IP-deficient cells show resistance to multiple chemotherapeutic agents due to the upregulation of WNT/β-CATENIN and, ultimately, the overexpression of the secreted heterodimeric isoform of the Clusterin (*CLU*) gene [95]. EMT might also play a pivotal role in CaP chemoresistance, and E-Cadherin is an important marker in the development of chemoresistance. E-Cadherin is downregulated in chemoresistant CaP cells, leading to the upregulated expression of Vimentin and Claudin-1. Moreover, the downregulation of E-Cadherin leads to the upregulated expression of the Notch signaling pathway [96]. Furthermore, zinc finger E-box-binding homeobox 1 (ZEB1), a key EMT activator, has been associated with Docetaxel resistance. The overexpression of ZEB1 is correlated with the expression of multidrug-resistance-associated protein 1 (MRP1) and ATP-binding cassette subfamily C member 4 (MRP4), a poor Docetaxel transporter. Moreover, the ZEB1-mediated upregulation of MRP4 is characterized by lower BCL2 levels and higher BAX levels and caspase 3 expressions, leading to decreased apoptosis [97]. Considering the immunological aspect, the JAK/STAT pathway is very important for the expression of PD-L1, which lets tumor cells escape cell death via a T-cell immune response [98]. Moreover, it was also observed by Xu et al. [99] that the antitumor pathways of natural killer cells may also be repressed through the expression of PD-L1 via NKDG2D ligands blocking the surface of the cells. In CRPC, natural killer (NK) cell-mediated cytotoxicity has inclined resistance, as PD-L1 and NKG2D ligand levels were dysregulated by IL-6. It was conferred that the JAK/STAT pathway blockage was shown to cause increased susceptibility to NK-cell-arbitrated death in CRPC cells [100].

Circular RNA Foxo3 is downregulated in high-grade CaP cells. The dysregulation of circular RNA Foxo3 has been implicated in CaP cell survival, metastasis, invasion, and Docetaxel chemoresistance. Circular Foxo3 sponges miRNA and MDM2, which represses parent Foxo3; thus, the repression of circular RNA Foxo3 results in the repression of Foxo3 mRNA, ultimately reducing the life span of tumor-bearing mice [101]. Adipose stromal cells are the progenitors of adipocytes that help in tumor growth and development. In human cell co-culture models, adipocyte stromal cells can induce EMT and chemoresistance against Docetaxel, Cabazitaxel, and Cisplatin chemotherapy in CaP cells. The inhibition of adipose stromal cells via the hunter–killer peptide D-CAN suppresses obesity-associated EMT, cancer progression, and Cisplatin chemoresistance [102].

In CRPC, Docetaxel induces autophagy by inducing the Beclin1-Vps34-Atg14 complex. Interestingly, Docetaxel-induced autophagy in CRPC cells inhibits the signal transducer and activator of transcription (STAT) 3 and promotes chemoresistance [103]. CSCs are a small population of cancer cells with the property to renew themselves and are thought to be involved in tumor heterogeneity and chemoresistance. NOTCH signaling pathways maintain the CSCs. In prostate CSCs, the intracellular domain of NOTCH that regulates various downstream genes involved in differentiation and self-renewal is higher than that in non-prostate CSCs [104]. Further, Maurya et al. [34] reported that the β-CATENIN /c-MYC axis along with c-FLIP promotes Enzalutamide resistance in both CaP and CSC cells. In addition, Du145/DTX50 and PC-3/DTX30, the Docetaxel-resistant cell line, were used to check out the response of Cabazitaxel. Parameters like the correlation analysis, which was used to screen the cross-resistance of the exposed cell, were also observed. It was recorded that the constantly increased expression level of kinesin family member 14 (KIF14) was recognized as the remarkable cause of cross-resistance in the exposed cell. It was also observed that silencing KIF14 expression would be helpful in restoring the sensitivity of resistance to CaP cells against Cabazitaxel and Docetaxel, leading to the decreased proliferation and increased apoptosis of resistant CaP cells. The increased expression of KIF14 promotes chemoresistance by inhibiting the phosphorylation of AKT pathways [105]. Newly emerged findings indicate that hypermethylation and somatic mutations in DNMT3B, TET2, IDH1, and B-RAF genes are present in 22% of tumors that are associated with castration-resistant prostate cancer (CRPC) [106]. Chemoresistance is one of the major setbacks for CaP drug development, and it is essential to find a drug that can overcome this hurdle.

## 5. Chemosensitization of Prostate Cancer with Phytochemicals

**Figure 3:**
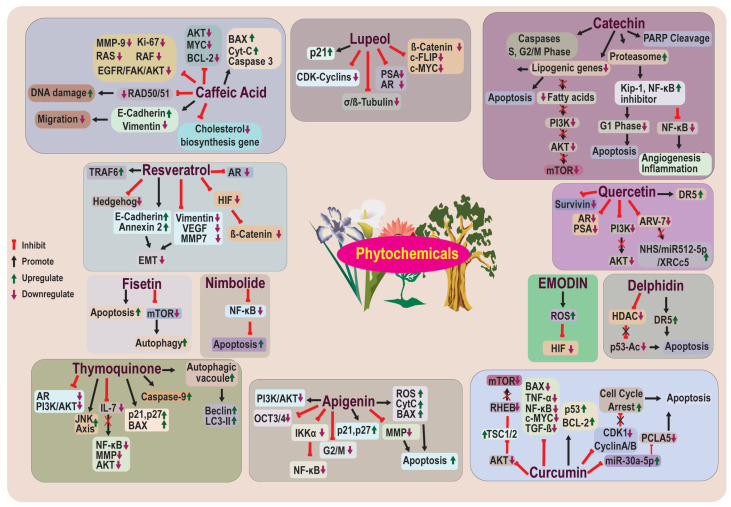
Chemosensitization of prostate cancer therapy resistance using phytochemicals. The phytochemicals isolated from different plants facilitate the sensitization of resistant cells for different anti-prostate cancer therapies by targeting various signaling molecules and pathways. AKT: Protein Kinase B; AR: Androgen Receptor; AR-V: Androgen Receptor Variant; BAX: Bcl-2-Associated X Protein; CDK: Cyclin-Dependent Kinase; CytC: Cytochrome C; DR5: Death Receptor 5; EGFR: Epidermal Growth Factor Receptor; FAK: Focal Adhesion Kinase; HDAC: Histone Deacetylase; IKK: Inhibitor of Nuclear Factor κB (IκB) Kinase; LC3: Microtubule-Associated Protein 1A/1B-Light Chain 3; MMP: Matrix Metalloproteinase; NF-kB: Nuclear Factor Kappa B; PCLAF: PCNA-Associated Factor; PI3K: Phosphatidylinositol 3 Kinase; PSA: Prostate-Specific Antigen; RHEB: Ras Homolog Enriched in Brain; ROS: Reactive Oxygen Species; TGF: Tumor Growth Factor; TNF: Tumor Necrosis Factor; TS: Tuberous Sclerosis Protein.

Plants are rich sources of bioactive compounds, and the secondary metabolites that are present in plants have shown excellent interspecies interaction, which can modulate the expression of various human proteins [107]. The use of phytochemicals as anticancer agents represents many bioactive compounds that have shown lesser side effects than conventional chemotherapeutic agents [108]. Phytochemicals have shown anticancer activity by targeting apoptosis, proliferation, invasiveness, and metastasis. Moreover, phytochemicals can increase the overall efficacy of clinically approved therapies, including radio- and chemotherapy [109]. Phytochemicals alone, or combined with other therapies or nanomedicines, can regulate various factors of the body to prevent or treat human CaP [110] (Figure 3). A positive correlation was suggested between an increased consumption of plant-based foods (cruciferous vegetables, whole grain bread, legumes, and vitamin C-rich vegetables) and a reduced CaP risk [109]. In this regard, we carefully describe the state-of-the-art concerning phytochemicals and their anticancer role in preventing, eradicating, and/or sensitizing CaP cells in the next section.

### 5.1. Curcumin

Curcumin or diferuloylmethane is naturally found in turmeric and exerts several biological efficacies through various mechanisms [111]. The anticancer properties of Curcumin have been explored since 1985, and various mechanisms have been proposed to understand the underlying mechanism of Curcumin [112]. Katta et al. [113] investigated the novel gene network and signaling pathways involved in Curcumin’s chemopreventive and anticancer effects against androgen-dependent non-metastatic and independent metastatic cell lines. It was observed that significant signaling pathways are altered at the genomic level, including the c-MYC and TGF-β signaling pathways. Moreover, it was observed that Curcumin has an anticancer effect on the androgen-sensitive 22rv1 and the LNCaP-derivative C4-2B CaP cell lines, and Curcumin was found to be effective in the sensitization of CaP cells for TRAIL-mediated immunotherapy [114]. Deeb et al. [115] reported that the combination of Curcumin with TRAIL-mediated immunotherapy sensitizes the CaP cell lines LnCaP, Du145, and PC3; induces apoptotis; and inhibits NF-kB expression. In an investigative study to elucidate the role of Curcumin, it was observed that Curcumin induces the expression of miR-30a-5p, a well-known tumor suppressor, which is inversely correlated with the expression of PCNA clamp-associated factor (PCLAF). Hence, Curcumin inhibits tumor progression, survival, and metastasis by regulating the miR-30a-5p/PCLAF axis [116].

In another study, it was found that the PC-3 and DU-145 organoids formed after three days of culture in ECM can be significantly reduced by the administration of different concentrations of Curcumin in a short span of time (10–12 days) by inducing apoptosis and reducing proliferation [117]. Although Curcumin may possess some drawbacks, such as a low bioavailability and absorption, various derivatives and nano-modification can negate the problem. Combining low-dose Curcumin and visible light irradiation can significantly reduce CaP tumor growth, adhesion, and invasion. It was observed that Curcumin plus light could trigger G2/M cell cycle arrest by inhibiting CDK1-Cyclin A/B, the AKT-mTOR pathway, and Integrin-α/β [118]. Moreover, aptamer-functionalized Curcumin and Cabazitaxel (2:5) co-delivered lipid–polymer hybrid nanoparticles can inhibit CaP progression and alleviate the anticancer therapy side effect as determined by the unchanged body weight and levels of ALT, LDH, and BUN parameters [119]. Furthermore, Curcumin–Phospholipion–Scorpion venom showed an increased anticancer effect on CaP cells by inducing cell cycle arrest at the G2/M and preG1 phases and altering the expressions of BCL-2, BAX, p53, Caspase, TNF-α, and NF-κB. Moreover, phospholipion also reduces mitochondrial permeability, making it a novel anti-CaP therapy [120].

### 5.2. Catechins

Catechins are the polyphenols (flavon-3-ol) that comprise about 40% dry weight of tea leaves and are identified to have therapeutic relevance against CaP progression. Catechins chemo prevents CaP progression by targeting multiple biochemical and molecular cascades involved in carcinogenesis. Catechins promote the accumulation of the p27 (Kip1) and NF-κB inhibitors by inhibiting the chymotrypsin-like activity of the proteasome and consequently arresting the cell cycle at the G1 phase. Further, NF-κB depletion induces cancer cell apoptosis and inhibits inflammation and angiogenesis [121]. Catechin extracts and nano-emulsions from oolong tea leaves exert their anticancer effects by targeting Caspase 9/8/3. Moreover, Catechin administration causes cell cycle arrest at the S and G2/M phases, significant decreases in the serum levels of EGF and VEGF, and reductions in the tumor volume and weight [122]. Epigallocatechin Gallate (the most abundant Catechin in green tea) inhibits CaP by promoting cancer cell death, suppressing the activation of agonist-dependent AR and its regulated genes [123]. Moreover, Epicatechin gallate significantly inhibits CaP progression by modulating lipid metabolism. The enhanced synthesis of fatty acids and upregulated lipogenic genes are typical for developing CaP cells. Epicatechin gallate inhibits de novo fatty acid synthesis via the downregulation of the expression of acetyl-CaA carboxylase, fatty acid synthase, and ATP citrate lyase. Moreover, Epicatechin gallate can inhibit the PI3K/AKT/mTOR signaling pathway [124]. When EGCG and Apo2L/TRAIL are used together, they create a more potent effect on LNCaP cells through enhanced apoptosis via upregulated PARP and Caspase cleavage and altered cell communication of DR4/TRAIL R1, Fas-associated death domain, and FLICE-inhibitory protein, making the cells more responsive to TRAIL [125].

### 5.3. Caffeic Acid

Caffeic acid and its naturally occurring derivatives, Caffeic acid phenethyl ester (CAPE), are phenolic acid compounds found in tea, wine, and coffee and have shown anticancer effects against various cancers both in vitro and in vivo [126]. The administration of CAPE to CaP cells sensitizes them to radiation therapy by enhancing the ionizing radiation-induced gamma H2AX foci and cell death. Moreover, CAPE and radiation therapy can induce DNA damage (decrease DNA repair proteins, RAD50 and RAD51) and decelerate the migration (increase E-Cadherin and decrease Vimentin expression) of CaP cells [127]. In another investigative study, it was observed that CAPE could sensitize CaP cells to Docetaxel treatment. A combination of CAPE and Docetaxel induces CaP cell deaths and suppresses cancer proliferation and progression by downregulating the expression of anti-apoptotic markers and caspase activation, Bcl-2, AKT-2, c-MYC, and pyruvate kinase M2. However, combining phenylether and chemotherapeutic drugs can increase the expressions of BAX, Caspase 3, cytochrome c, and glucose-6-phosphate dehydrogenase. Further, CAPE-Docetaxel treatment interferes with DHCR24 (cholesterol biosynthesis genes), lanosterol synthase, and genes involved in glycolysis and the TCA cycle [128]. Tseng et al. [129] found that the administration of CAPE (15 mg/kg) every 3 days for 14 days can significantly suppress CaP growth by suppressing the expressions of Ki67, MMP-9, p-AKT, Ras, and Raf in xenograft mice. Also, CAPE modulates EGFR/FAK/AKT signaling, further decreasing CaP cell migration.

### 5.4. Thymoquinone

Thymoquinone is a phytochemical that is commonly found in the volatile oil of black cumin seeds (*Nigella sativa)* and exerts anticancer and anti-inflammatory effects [130]. Docetaxel toxicity and resistance are common side effects of the CaP-treating chemotherapeutic drug. Thymoquinone showed a synergistic effect, in combination with Docetaxel, on CaP cells. The combination treatment showed increased cytotoxicity and apoptosis. Moreover, Thymoquinone administration increased the sensitivity of CaP cells to Docetaxel by modulating apoptotic markers [131]. Further, Thymoquinone induced the formation of autophagic vacuoles and increased the expressions of Beclin-1 and LC3-II protein [132]. The interaction of Interleukin (IL)-7 with its receptor plays a significant role in CaP metastasis. Thymoquinone inhibits tumor progression and metastasis by suppressing IL-7 expression. Further, Thymoquinone also downregulates the expressions of IL-7-induced p-AKT, NF-κB, and MMP3/7 in a dose-dependent manner [133]. Thymoquinone can inhibit the viability of CaP cells by increasing the expression of Caspase 9 and can decelerate CaP growth by activating the JNK signaling pathway, cell cycle arrest, DNA damage, apoptosis, and oxidative cytotoxic DNA damage [134]. Also, Thymoquinone represses the nuclear localization of AR and suppresses the AR-directed transcriptional activity in CaP cells. Moreover, Thymoquinone downregulates E2F-1 and upregulates p21, p27, and BAX expressions. The combination of Thymoquinone with Docetaxel synergistically induces apoptosis and inhibits PI3K/AKT signaling in CaP cells [135].

### 5.5. Quercetin

Quercetin is a bioactive compound that is commonly found in nuts, teas, and vegetables and has various anticancer activities. Quercetin can impede the initiation and progression of CaP by discursively blocking the promoters of AR and PSA genes. Moreover, Quercetin has shown differential effects on normal and CaP cells, which suggests the specific cytotoxic effect of Quercetin on CaP cells [136]. Ghafouri-Fard et al. [136] illustrated the possible mechanism by which Quercetin modulated the signaling cascades involved in CaP. Quercetin and Docetaxel hinder CaP tumor growth, as Lu et al. [137] observed in xenograft tumors. A stronger activation of AR and PI3K/AKT has observed that Docetaxel-resistant CaP subclones and Quercetin application can sensitize CaP cells to chemotherapy by inhibiting the PI3K/AKT pathway and promoting apoptosis. Quercetin radiosensitized CaP cells in another study by targeting radiation-induced AR variant 7-mediated circular NHS/miR512-5p/XRCC5 signaling [138]. Moreover, Quercetin also reverses Docetaxel resistance in CaP cells. Also, Quercetin inhibits migration, proliferation, colony formation, invasion, and apoptosis in CaP [137]. The induction of apoptosis in prostate cancer cells by TRAIL was amplified when combined with quercetin. This combination led to a heightened expression and stability of DR5 (Death receptor 5), reduced levels of Survivin via the ERK-mediated deacetylation of H3 histone protein, and decreased AKT phosphorylation, contributing to the enhanced apoptotic response [139].

### 5.6. Lupeol

Lupeol is a bioactive compound of the triterpenoid class that is commonly found in olive, mango, aloe vera, etc., and has antimicrobial, antioxidant, and anticancer properties [140]. Lupeol exerts an anti-neoplastic effect on CaP cells by decreasing the serum PSA levels. Moreover, Lupeol alters AR, Cyclin D1, and MMP-2 expressions; regulates β-CATENIN, GSK-3β-axin complex, and TCF elements; and inhibits tumorigenicity in androgen-dependent and castration-resistant phenotypes [70,141]. Siddique et al. [141] also observed that Lupeol significantly inhibits the transcriptional function of AR and PSA expression by competing antagonistically with androgen for the AR. Furthermore, Lupeol induces cell growth arrest by decreasing the levels of Cyclins A/B1/D1/D2/E2 and CDK-2 and increasing the expression of p21 (CDK inhibitor). Also, Lupeol regulates the activity of β-CATENIN, α-tubulin and β-tubulin (components of microtubules), Stathmin (microtubule regulatory protein), Survivin (pro-survival protein), and c-FLIP [142,143]. Lupeol chemosensitizes CaP cells for Enzalutamide treatment by targeting β-CATENIN, c-FLIP, and c-MYC [34].

### 5.7. Apigenin

Apigenin, a dietary flavonoid that is abundant in various plants like parsley, celery, celeriac, basil, chamomile tea, and kumquat-like fruits and vegetables offers multiple health benefits, including anticancer properties [110]. Various studies have suggested the anticancer role of Apigenin against CaP cells. Moreover, Apigenin represses CaP CSCs by significantly increasing the expressions of p21 and p27 and represses CaP proliferation by inducing G2/M cell cycle arrest [144]. It was observed that Apigenin inhibits IKKa, leading to the inhibition of NF-kB, and consequently suppressing CaP progression. Apigenin induces ROS-dependent apoptosis in CaP cells. Apigenin was observed to significantly disrupt mitochondrial membrane potential via both transcriptional-dependent and independent p53 pathways. Further, Apigenin promotes Cytochrome C release, increases Caspase-3 cleavage, and upregulates BAX in favor of CaP cell apoptosis [145]. Apigenin was also found to inhibit the activity of histone deacetylase (HDAC) in CaP cells, enhancing the acetylated level of Ku70 and subsequent dissociation from BAX, thus reversing CaP and promoting epigenetic changes [146]. In another study, Apigenin was observed to exert cytotoxic effects on CaP and CSCs by increasing p21 and p27. Further, Apigenin upregulates Caspase-8, -3, and TNF-α and promotes extrinsic caspase-dependent apoptosis in CaP CSCs. Interestingly, in CaP cells, Apigenin promotes the intrinsic apoptotic pathway by upregulating BAX, Cyt-C, and Caspase-3. Moreover, Apigenin targets the PI3K/AKT/NF-kB signaling nexus and downregulates the OCT3/4 pluripotency markers [147].

### 5.8. Genistin

Genistin is a soy phytoestrogen with known anticancer activity. CaP cells activate DNA repair pathways in response to certain chemotherapeutic agents, leading to drug resistance. Genistin sensitizes CaP cells towards various chemotherapeutic agents by targeting DNA repair enzymes such as apurinic/apyrimidinic endonuclease 1 (APE1). Further, Genistin can demethylate estrogen receptor (ER)-β in a dose-dependent manner, thereby repressing the tumor-promoting effect of methylated ER-β [144]. Genistin was observed to exert an antioxidant effect on CaP cells by increasing the expression of the glutathione peroxidase enzyme inside the cell, which further resulted in decreased oxidative-stress-mediated DNA damage. Intriguingly, the concomitant administration of Genistin with Cholecalciferol before surgery was reported to decrease the growth of CaP cells [148]. Genistin was also observed to inhibit CaP proliferation in wild AR-expressing cells by repressing AR nuclear localization in a dose-dependent manner. However, Genistin showed biphasic results at the physiological concentration by activating mutant ARs and increasing AR expression [149].

### 5.9. Delphinidin

Delphinidin, an anthocyanidin made CaP cells more responsive to TRAIL-induced apoptosis by promoting the expression of DR5. Furthermore, they triggered apoptosis in LNCaP cells by inducing p53 acetylation through the inhibition of HDAC activity. Additionally, the administration of Delphinidin significantly restrained the growth of PC-3 xenografts in athymic nude mice [150].

### 5.10. Resveratrol

Resveratrol, also known as 3,5,4′-trihydroxy-trans-stilbene, is classified as a stilbenoid within the polyphenol family. It is characterized by its molecular structure, which consists of two phenol rings connected by an ethylene bridge. Resveratrol can inhibit CaP cell proliferation by blocking AR signaling through the inhibition of AR agonists, receptor activity, and the expression of the AR target gene. Resveratrol also inhibits PI3K activity and enhances the functional activity of PTEN [151]. Another study reported that Resveratrol can inhibit castration-resistant and -sensitive CaP cells by inhibiting Hedgehog signaling and alleviating the tumor necrosis factor receptor-associated factor 6 (TRAF6). Further, Resveratrol inhibited Vimentin, VEGF, and MMP7 expression while stimulating the expressions of E-Cadherin and Annexin 2 to inhibit EMT and CaP progression [152]. Resveratrol reduces CRPC tumor progression by repressing β-CATENIN-mediated AR signaling and inhibiting the nuclear translocation of β-CATENIN through the suppression of the HIF-1 expression level. Clinical studies have shown that Resveratrol is safe to be administered at a dose as high as 4000 mg/day, and its chemopreventive activity can be studied in conjunction with other naturally occurring antioxidants [153]. Although various studies have explored the chemopreventive and chemosensitizing effects of Resveratrol, the biological activity of the phytochemical is limited due to metabolic instability and photosensitivity. Resveratrol analogs and nanoparticles can be utilized to enhance the efficacy of the natural compound. Badawi [154] reviewed that Resveratrol-loaded nanoparticles showed exemplary anticancer activity against both therapy-sensitive and -resistant CaP. He observed that Resveratrol nanoparticles showed synergistic effects with Docetaxel and increased the efficacy of chemotherapy. HS-1793 is a novel analog of Resveratrol, which has shown an anticancer effect on the resistant CaP cell line, PC3, by decreasing the expression of HIF-1 in a time-dependent manner. Further, HS-1793 also reduced the expression of VEGF through the inhibition of PI3K phosphorylation and AKT and promoted the proteasomal degradation of the HIF-1 α protein [155].

### 5.11. Fisetin

Fisetin, also known as 3,3′,4′,7-tetrahydroxy flavone, is a bioactive flavonol compound that is naturally found in strawberries, apples, persimmons, grapes, onions, and cucumbers. Fisetin exhibits a range of beneficial properties, including anticancer effects [156]. Fisetin enhanced the chemotherapeutic efficacy of Cabazitaxel under both androgen-dependent and -independent conditions. It was observed that a combination of Fisetin and Cabazitaxel can result in reduced toxicity and the synergistic deceleration of therapy resistance, invasion, and migration of CaP cells [156]. Fisetin enhanced the susceptibility of DU 145, LNCaP, and PC-3 cells to TRAIL-induced apoptosis by activating receptor-mediated mitochondrial apoptotic pathways. Additionally, Fisetin triggered autophagic cell death by suppressing mTOR and PI3K/Akt signaling [150].

### 5.12. Other Phytochemicals

Nimbolide is a triterpene compound derived from the leaves and blossoms of the neem tree and has an anticancer effect. Nimbolide can enhance the cytotoxic effect of Docetaxel and sensitize CaP cells for chemotherapy. The combination of Nimbolide with Docetaxel can decrease CaP cells’ viability and induce apoptosis via the abrogation of therapy-induced NF-kB and its effect on the downstream anti-apoptotic factors [157]. Emodin is a phytochemical that is found in the roots and rhizomes of various plants such as *Aloe vera*, *Rheum palmatum*, and *Cassia obtusfolia* and fungal species such as *Aspergillus.* Emodin showed a synergistic anticancer effect with Cisplatin against CaP cells by suppressing multidrug resistance factor and HIF-1 through ROS generation [158]. Sulforaphane is present in foods primarily in the form of the glycoside glucoraphanin, which belongs to the isothiocyanate group. Studies have revealed that sulforaphane enhances the antiproliferative and proapoptotic impacts of TRAIL in CaP models. Sulforaphane exhibits cytotoxic effects on both androgen-independent PC-3 cells and androgen-dependent LNCap cells. When combined with sulforaphane, TRAIL’s cytotoxic effects are intensified in PC-3 models, and it sensitizes TRAIL-resistant LNCaP cells to TRAIL. Recent research underscores sulforaphane’s ability to sensitize prostate cancer cells to TRAIL-induced apoptosis. TRAIL, known for its selective toxicity against malignant cells, is regarded as a promising anticancer agent [159]. Ocoxin is a natural nutritional blend that can serve as a complementary option alongside existing antitumor treatments like Docetaxel, Enzalutamide, and Olaparib (PARP inhibitor). Its composition includes extracts from green tea (EGCG), licorice (EGCG and Glycyrrhizin), cinnamon (Catechins and Quercetin), and various other natural ingredients [160].

The use of phytochemicals in cancer treatments is increasing globally, and scientific communities are exploring the anti-neoplastic effects of phytochemicals [108]. Various phytochemicals with various pharmacological health benefits are explored for their possible anticancer effect against CaP cells. However, bioavailability and solubility are the major hurdles in implementing these phytochemicals in therapeutic management. Many new strategies are being developed to improve the compromised bioavailability of these phytochemicals. Wide pharmacological benefits can be exploited soon by developing nano-based alternatives or artificially synthesized derivatives of phytochemicals with increased bioavailability and solubility. Also, identifying propitious effects and underlying mechanisms of action should be explored to advance drug development.

## 6. Conclusions and Future Perspectives

CaP is a prevalent and substantial health concern among men across the globe. It involves a range of pathways that play roles in its emergence, advancement, invasion, migration, and resistance to chemotherapy. While early-stage CaP patients benefit from androgen suppression therapy, the development of biochemical recurrence and castration-resistant CaP remains frequent. Despite several available drugs and hormone-based treatments for CaP, the survival rates continue to remain disappointingly low. To make significant progress in treating CaP patients, it is imperative to delve deeper into the molecular mechanisms underlying its initiation, progression, and cellular reprogramming. Compared to the extensive pre-clinical research conducted on phytochemicals, our knowledge from clinical trials remains relatively sparse. To propel this field forward, comprehensive investigations across all phases of clinical trials are imperative. Further, many phytochemicals showed reduced bioavailability and solubility, which is one of the main reasons why phytochemicals cannot succeed in clinical trials. Finding alternatives, chemically synthesized derivatives, and nano-derivatives can increase the beneficial efficacy of phytochemicals. Additionally, exploring the potential of natural compounds (phytochemicals) in preventing and sensitizing cancer cells to chemotherapy holds promise. This avenue of research could offer enhanced therapeutic outcomes for individuals battling CaP.

## Data Availability

Not applicable.
